# Limb salvage surgery for calcaneal chondrosarcoma: A case report

**DOI:** 10.1097/MD.0000000000031578

**Published:** 2022-12-23

**Authors:** Jingming Wang, Xiuchun Yu, Kai Zheng, Ming Xu

**Affiliations:** a Department of Orthopaedics, The 960th Hospital of Chinese People’s Liberation Army, Jinan, China.

**Keywords:** calcaneal sarcomas, reconstruction, total calcanectomy

## Abstract

Limb salvage surgery for calcaneal sarcomas remains challenging due to its poor compartmentalization. While below-knee amputation is still the standard choice of operative treatment, total calcanectomy with or without reconstruction was advocated. This report aims to analyze the clinical outcome of calcaneal reconstruction with cement and replantation in situ after the inactivation of tumor.

**Methods:**

We describe a 73-year-old male patient who suffered chronic pain and increasingly larger neoplasm in the left foot for about 3 years. Based on the results of percutaneous biopsy, a diagnosis of chondrosarcoma was made.

**Results:**

The patient underwent total calcanectomy, inactivation of calcaneus tumor, and reconstruction with cement. The Achilles tendon was detached through a Cincinnati incision. No adverse events occurred both during and after the surgery. At the last follow-up of 29 months, the patient claimed no pain, no evident limp, or any limitation of daily activities. Image examination, weight-bearing test, and MSTS score revealed a satisfactory result.

**Conclusion:**

Calcaneal reconstruction with bone cement after total calcanectomy, inactivation of calcaneus tumor, and replantation in situ is likely to provide a feasible surgical choice and a satisfactory clinical outcome.

## 1. Introduction

Primary malignancies located in the calcaneus are extremely rare, accounting for less than 1% of the primary tumors of the skeleton, whereas the most common are osteosarcoma, chondrosarcoma, and Ewing’s sarcoma.^[1]^ Due to this low incidence, to the best of our knowledge, there are only 15 reports discussing 35 patients with calcaneal malignancies, including case reports and a series of cases with surgical options, and the clinical outcomes are not well known.

Adequate local control is one of the most important factors in the treatment of primary malignant bone tumors. Amputation is the most common surgical option for achieving local control, as it is not technically demanding. However, the result of this, which is limb loss, will cause chronic pain, mental sickness, and other complications.^[2]^

Total excision of the calcaneus is the primary treatment of choice for malignant tumors. Only one side of the bone is close to the major neurovascular structure, which can be preserved in most patients to provide safe surgical margins.^[3]^ Reconstruction of the irregular shape and weight-bearing function of the calcaneus is challenging and technically challenging. Various reconstructive techniques with allografts, autografts, or prostheses have been proposed,^[3–18]^ and none have reported the reconstruction of the inactivated calcaneus and replantation in situ.

## 2. Case presentation

**Figure 1.**
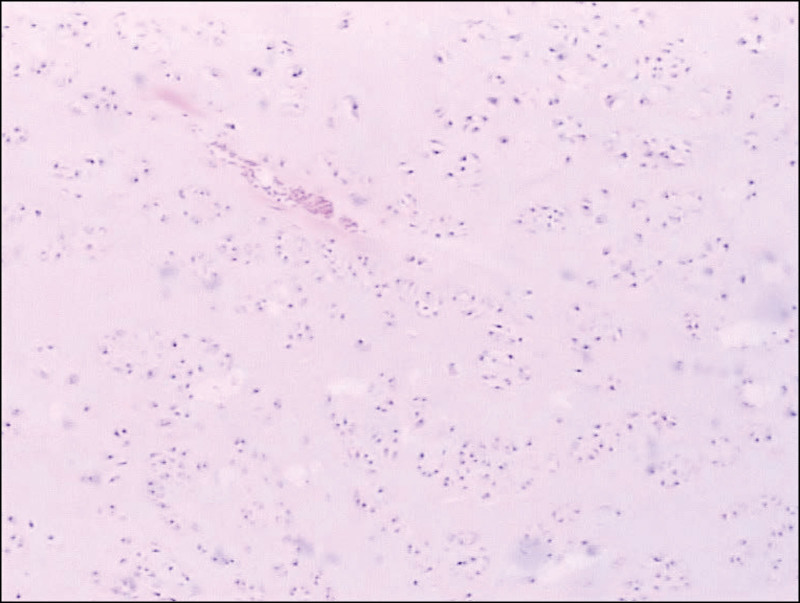
Biopsy revealed chondrosarcoma which is intermediate grade (grade 2). The histologic section reveals cells with bizarre and monstrous nuclei. nuclei were round and centrally located, with a prominent central nucleolus, surrounded by abundant eosinophilic cytoplasm. (HE, 40×).

**Figure 2.**
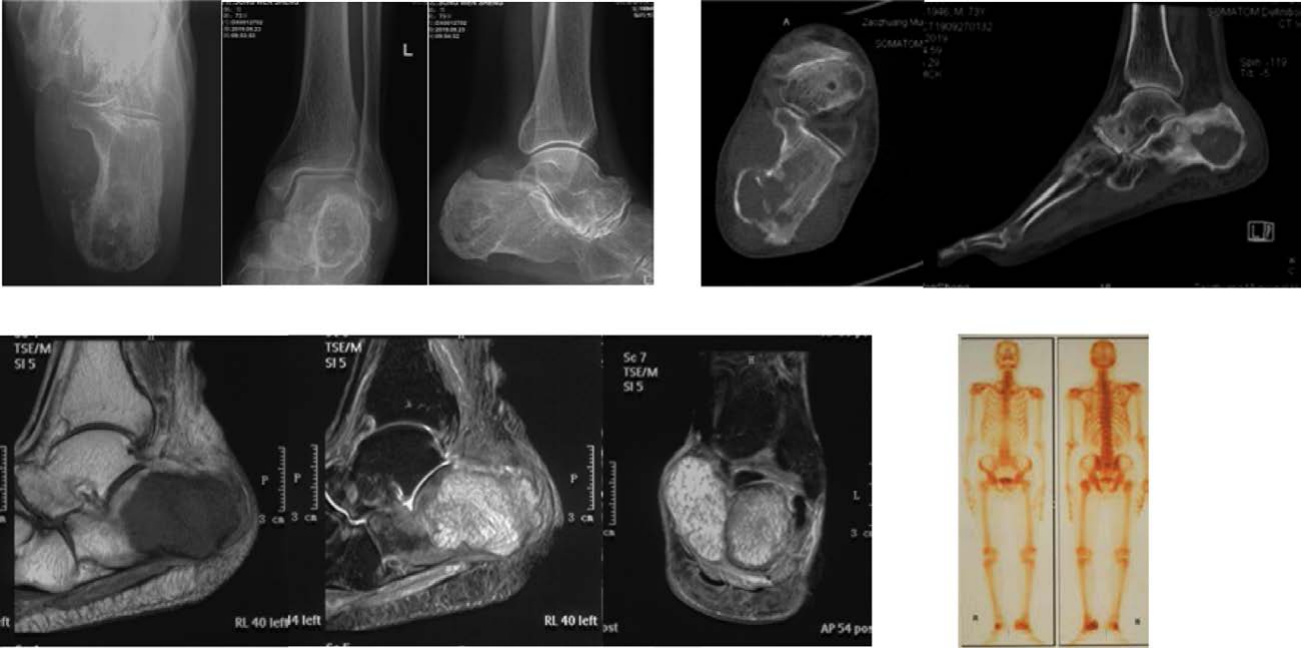
A 73-year-old male patient. X-ray show a radiolucent lesion occupying the left calcaneous. CT and MRI show some involvement of the surrounding soft tissue, while ECT showed intense activity in the calcaneus with no other sites of abnormal tracer absorption. CT = computed tomography, ECT = emission computed tomography, MRI = magnetic resonance imaging.

**Figure 3.**
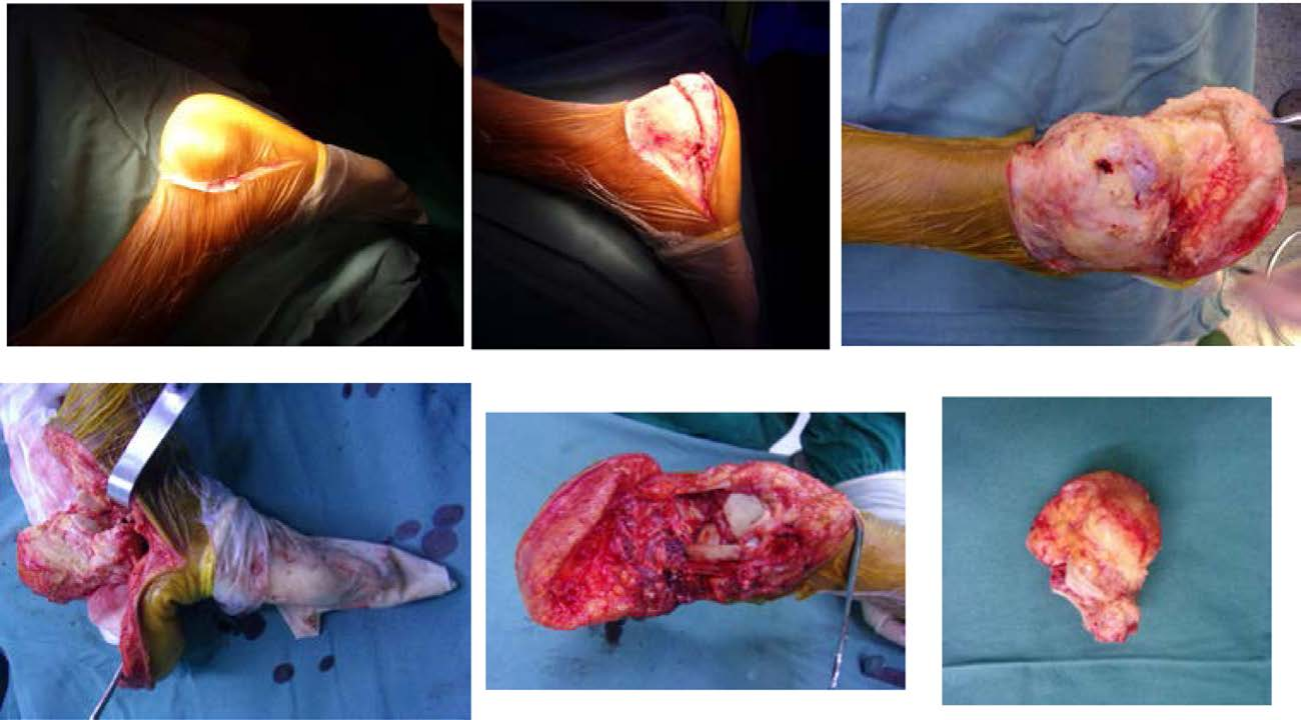
A total calcanectomy was performed with the patient using a Cincinnati incision.

A 73-year-old male patient with chronic pain and an increasingly large neoplasm in the left foot for approximately 3 years was admitted to our institution in October 2019. Percutaneous biopsy revealed chondrosarcoma (Fig. 1). CT and MRI showed some involvement of the surrounding soft tissue, while emission computed tomography showed intense activity in the calcaneus, with no other sites of abnormal tracer absorption (Fig. 2). The patient underwent total calcanectomy and reconstruction with inactivated calcaneus and bone cement. The operation was performed with the patient in the prone position using a Cincinnati incision (Fig. 3). While one surgeon debrided the articular cartilage from the inferior articular surface of the talus and posterior surface of the cuboid, the other surgeon derided the articular cartilage from the calcaneus. The calcaneus was inactivated using ethanol (30 min). Using a high-speed burr, a groove was created on the inferior surface of the talus to accommodate the graft. Under fluoroscopic control, the graft was fixed to the talus and cuboid using multiple cannulated titanium screws to obtain a construct resembling talocalcaneal and calcaneocuboid arthrodesis. A bone cement was used to fill the calcaneus. The triceps surae were reconstructed using a nonabsorbable suture of the Achilles tendon to the bone graft after drilling a hole into the posterior part of the graft.

The drainage tube was removed when fluid drainage was less than 20 mL at 1 week, and the incision sutures were removed 2 weeks after surgery. A postoperative below-knee plaster cast was used for 8 weeks. Subsequently, he began progressive passive and active movement of the ankle without weight for one additional month. Partial weight-bearing with crutches was allowed at 15 weeks after surgery and full weight-bearing at 6 months when clear radiographic signs of union were evident. Patients were followed-up with periodic clinical and radiological examinations. CT scans were performed annually to detect metastatic diffusion. Functional results were evaluated according to the system proposed by the International Society of Limb Salvage and approved by the Musculoskeletal Tumor Society.^[19]^

**Figure 4.**
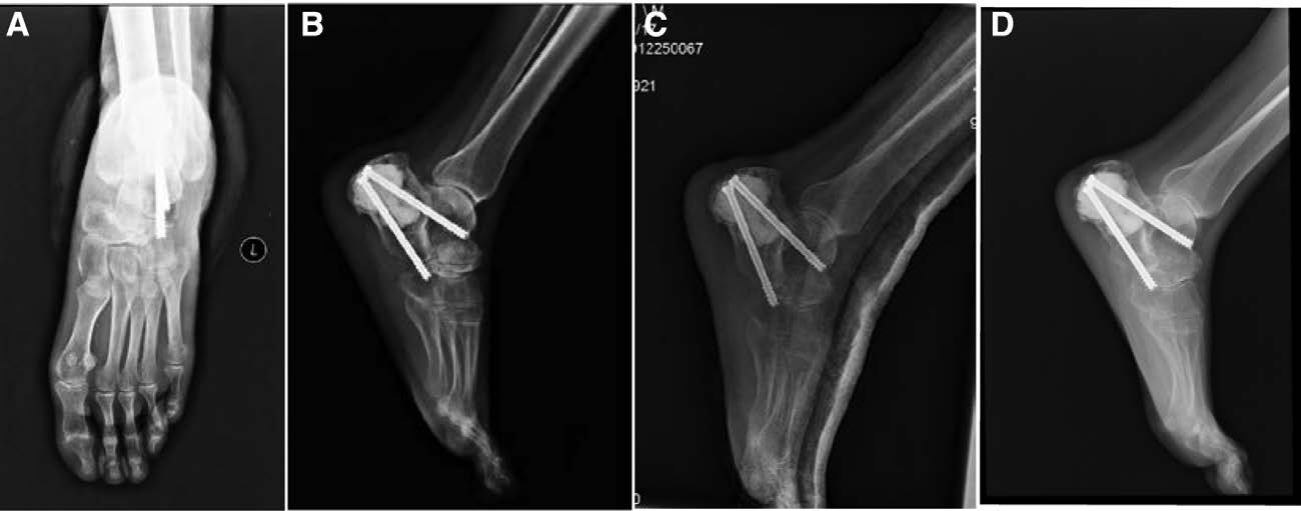
The arthrodesis healed successfully without fixation failure in 2 years of follow-up. (A, B) Anteroposterior and lateral X-ray images 7 days after surgery. (C) Lateral X-ray image 8 weeks after surgery. (D) Lateral X-ray image 8 months after surgery, the arthrodesis healed successfully.

**Figure 5.**
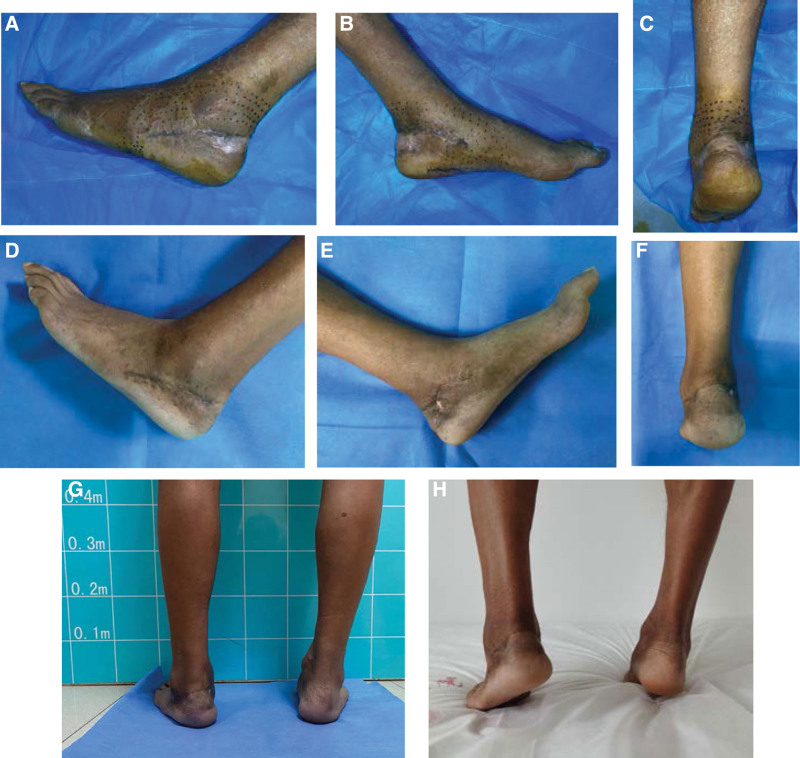
(A–C) Regional sense lost was found around the incision and heel at 8 weeks follow-up. (D–F) Partial weight-bearing with crutches was allowed at 15 weeks from surgery, the range of motion of the ankle was approximately normal. (G–H) The patient could bear full weight without support. A slight asymmetry between the 2 heels was observed with an almost normal gait at 8 months follow-up.

The flap survived without any complications. The arthrodesis healed successfully without fixation failure. No signs of metastasis or local recurrence were observed during the final follow-up. At the latest evaluation, the pain was completely absent, with no evident limb or limitation in daily activities. No special shoes were required for the patients. The patient was satisfied with the clinical results. The functional evaluation scores of the patients according to the ISOLS-MSTS system are shown in Table 1. A slight asymmetry between the 2 heels was observed with an almost normal gait. The patient was active in normal activity without any limitation, and active plantar flexion and dorsiflexion were observed (Figure 4 and 5).

**Table 1:** Patient`s demographics and outcomes.

No.	Age	Gender	Histopathology	Adjuvant therapies	Follow-up (mo)	MSTS score system (lower limb)						
						Pain	Function	Emotional	Supports	Walking	Gait	Final patient score of functional evaluation (%)
1	73	Male	Chondrosarcoma	N	29	5	5	5	5	5	4	29 (97%)

## 3. Discussion

Calcaneal malignancies are rare, accounting for approximately 1% of primary skeletal tumors and 2% of all skeletal metastasis.^[1]^ To date, 15 reports of 35 patients with calcaneal malignancies who underwent total calcanectomy have been published worldwide.^[3–18]^ Data from the 36 patients, including the present case, are reviewed in Table 2. Chondrosarcoma, osteosarcoma, and Ewing sarcoma are the most common malignant bone tumors of the calcaneus.^[20]^ There are no specific clinical features of calcaneal tumors, and the most common clinical symptoms are pain, edema, an enlarging mass, and loss of function.

**Table 2:** Review of the literatures.

Author	Year	No.	Sex	Age	Characteristics of the calcaneal lesion	Replacement	Clinical outcmome	Last follow-up
Prothesis								
Chou	2007	1	Female	31	Giant cell tumor	Polymethylmethacrylate	Chronic moderate pain, weight-bearing limitation, a mild antalgic gait without shoes	12 yr
Imanishi	2015	1	Male	71	Chondrosarcoma	3D printing calcaneal prosthesis	Full weight bearing	5 mo
No reconstruction								
Madhuri	2012	1	Male	6	Primitive neuroectodermal tumor	None (Attachment of Achilles tendon to talus)	Full weight bearing	32 mo
Ichiro	2018	1	Male	59	Metastasis of renal cell carcinoma	No bony reconstruction	Chronic moderate pain, weight-bearing limitation	1 yr
Allograft								
Li	2012	1	Male	36	Chondrosarcoma	Allograft and vascularized osteocutaneous fibular grafts	Revision surgery in 2 patients because of skin flap necrosis and infection. Full-weight bearing at 7 mo	24.5 mo
		1	Male	34	Fibrosarcoma			
		1	Female	27	Aggressive osteoblastoma			
		1	Male	32	Giant-cell tumor			
Wozniak	2007	1	Male	15	Chondrosarcoma	Allogenic femoral head and neck graft	Some limitation of the range of motion of the ankle. Full weight bearing.	9 yr
		1	Male	15	Synovial sarcoma			
		1	Female	16	Ewing sarcoma			
Muscolo	2000	1	Male	14	Chondrosarcoma	Allogenic fresh-frozen calcaneus was inserted	Some limitation of the range of motion of the ankle. No pain. Full weight bearing.	32 yr
		1	Male	41	Giant-cell tumor	Allogenic fresh-frozen calcaneus	Minor pain after strenuous walking. Some limitation of the range of motion of the ankle.	9 yr
Ayerza	2016	1	Male	21	Giant cell tumor	Total calcaneal allograft	1 deep infection, 1 allograft fracture, 1 nonunion. Full weight bearing.	5 yr
		5	Not mentioned	Not mentioned	Not mentioned			
Benjamin Degeorge	2016	1	Not mentioned	58	Chondrosarcoma	Calcaneus allograft	He was prescribed pronation insoles for daily use over a walking distance of 500 m.	1 yr
Ottolenghi	1953	1	Male	14	Chondrosarcoma	Calcaneal autograft and iliac autograft	Nonunion	32 yr
Autograft								
Kurvin	2008	1	Female	27	Osteosarcoma	Free iliac flap	Wearing an orthopedic over-the-ankle boot with special hindfoot cushioning. Little remaining pain.	8 mo
Yan	2010	1	Male	10	Osteosarcoma	Free iliac flap	Wound dehiscence	36.4 mo
		1	Male	15	Osteosarcoma			
		1	Male	19	Osteosarcoma			
Li	2010	1	Male	37	Chondrosarcoma	Pediculated fibular flap	No evident limp or limitation of daily activities in 4 patients and mild limp in 1 patient. Hematoma in 1 patient and skin margin necrosis in 1 patient (healed by debridement and delayed skin-grafting)	50.4 mo (32–76 mo)
		1	Female	43	Chondrosarcoma			
		1	Male	19	Osteosarcoma			
		1	Male	16	Ewing sarcoma			
		1	Female	23	Ewing sarcoma			
Kamal	2016	1	Male	46	Giant-cell tumor	Femoral head allograft and soft tissue coverage with sural flap	Full weight bearing	1 yr
Innocenti	2019	1	Female	18	Osteoblastoma	Free iliac crest flap	Displaced marginal fracture of a small posterior portion of the graft at 6 mo (conservative treatment)	19 yr
		1	Male	30	Osteoblastoma		Breaking one of the anterior screw (conservative treatment)	17 yr
		1	Male	18	Ewing`s sarcoma		Wound dehiscence (healed by negative-pressure wound therapy)	9 yr
		1	Female	42	Osteosarcoma		Wound dehiscence (healed by secondary intention)	6 yr
Our study	2022	1	Male	73	Chondrosarcoma	Bone cement	Some limitation of the range of motion of the ankle. No pain. Full weight bearing.	29 mo

Amputation is most commonly considered the only remaining option to achieve local tumor control and a good overall prognosis for patients with calcaneal malignancy.^[21,22]^ It is still the main choice of surgeons.^[23,24]^ However, chronic pain is highly prevalent in individuals with limb loss, regardless of the time since amputation.^[2]^

With its specialized anatomy, the foot is an indispensable component of normal ambulation. The mainstay of any reconstruction is to achieve stable conditions of the weight-bearing surfaces capable of withstanding pressure and shearing. In addition to the quality of the bone and soft tissue coverage, functional reconstructions of the foot should restore active and powerful extension (dorsiflexion) or plantar flexion to avoid putting as much weight on the heel as possible.

Advances in oncological treatment modalities and wide resection have made limb salvage increasingly possible. Total calcanectomy without reconstruction has been reported in 2 cases.^[6,7]^ Total calcanectomy is an effective method for the treatment of high-grade resectable tumors of the calcaneus, particularly in children. The Achilles tendon was then sutured to the posterior aspect of the talus. Madhuri reported the case of a 7-year-old child who underwent chemotherapy followed by total calcanectomy for a primitive neuroectodermal tumor of the calcaneum without reconstruction; the patient had near-normal function at 32 months follow-up. He believed that reconstruction after calcaneal excision is not warranted in every pediatric case.^[6]^ Ichiro reported the case of a 59-year-old man who underwent total calcanectomy for metastasis of renal cell cancer in the calcaneus without reconstruction. The patient complained of pain in the midfoot and could walk without aid for less than 600 m.^[7]^ Loss of the heel causes appearance deformities and unsatisfactory function. Compensation was achieved by placing a heel lift in the shoe. Total calcanectomy without reconstruction may not be an alternative treatment option for adults.

Thus, a reconstructive procedure may be attempted to provide the patient with the best functional and durable results after calcanectomy. Previous studies have reported the main reconstruction techniques for the hindfoot after total calcanectomy, including allograft, distally pedicled osteocutaneous fibular flap, vascularized iliac flap, and 3D-printed calcaneal prosthesis, with good functional outcomes.^[3–5,8–18]^

Chou reported the use of a polymethylmethacrylate calcaneal prosthesis, which caused chronic moderate pain and weight-bearing limitation, and she had a mildly antalgic gait without shoes.^[4]^ Imanishi reported the use of a 3D-printed titanium calcaneal prosthesis in a 71-year-old calcaneal chondrosarcoma patient. Although the range of motion of the right ankle remained restricted mainly in inversion and eversion, the patient was free of pain without medication and could walk unsupported on bare feet at 5 months follow-up.^[5]^

The allogenic femoral head and neck graft and allogenic fresh-frozen calcaneus were reported to be used in 7 cases of primary malignant tumors of the calcaneus. Good functional results were achieved at a long follow-up, and one patient had minor pain after strenuous walking. Meanwhile, secondary osteonecrosis of the hindfoot was observed at the 4-year follow-up.^[9,10,12,13]^ Li reported a series of 4 patients with primary calcaneal tumors who underwent total calcanectomy and reconstruction with the use of a composite of allograft and vascularized osteocutaneous fibular grafts. Revision surgery was necessary for 2 patients because of complications (skin flap necrosis and infection).^[8]^

A total of 14 patients who underwent free iliac flap or fibular flap surgery following total calcanectomy were reported in 5 articles.^[14–18]^ Reconstruction with a vascularized iliac crest flap presents many advantages; no bone donor site other than the iliac bone comes close to the required graft size for total restoration of the calcaneum. In addition, compared to the rib bone, it offers many more possibilities for bone and tendon fixation through its greater volume. Vascularized transplants have low resection morbidity, are very adaptable, and long vessels allow for a stress-free anastomosis. Moreover, if necessary, an osteofasciocutaneous flap can also be harvested from this anatomical site, enabling the simultaneous reconstruction of bone and soft tissue defects.^[25]^

However, allografts, autografts, and prostheses are not suitable for simultaneous bone and soft tissue coverage, donor site morbidity, and functional recovery. Incision complications were the main concern of clinical surgeons; wound dehiscence occurred in half of the patients and needed negative pressure wound therapy or secondary intention in previous reports.^[11,18]^ No incision complications occurred in our patient, and prophylactic antibiotics, drain placement, and careful attention to wound closure were standard clinical protocols to reduce the risk of surgical site infection. In addition, we recommend the Cincinnati incision because it provides adequate exposure to the calcaneus and forms unsightly scars. If skin flap necrosis is found, high-pressure oxygen might be a favorable method to improve the blood supply to the wound.

Li reported 3 cases of calcaneal tumors that had local recurrence because of initial curettage procedures in another hospital.^[8]^ The following is our experience with preventing local recurrence.

First, surgery to treat calcaneal malignancy should be performed by experienced oncologists. Second, complete inactivation and wide resection are the primary surgical principles. Finally, after the pre-installed screw channel was completed, cement was inserted, internal fixation was performed, and the procedure was not in the wrong order.

Selecting the appropriate treatment for bone defect reconstruction is challenging for surgeons, and biological reconstruction may be the optimal method for reconstruction. The alcohol-inactivated method has the advantages of effectively killing tumor cells, being more economical and convenient, and yielding the same shape for reimplantation.^[26]^ The disadvantage is that a long period is required to achieve bone union with the surrounding bone tissue. We observed union at 8 months follow-up, similar to previous studies.^[5,16]^

To our knowledge, there have been no reports of cases treated with total calcanectomy with in situ reconstruction after inactivation of the calcaneus. This is a promising way to treat calcaneal malignancy as a limb salvage procedure that provides good quality of life.

## 4. Conclusions

Calcaneal reconstruction with inactivated calcaneus is likely to provide good chances of a long-lasting result and fewer long-term complications in a weight-bearing area. Maintenance of the original heel can guarantee a better quality of life and function.

## Author contributions

WJM: Data collection, Manuscript writing. YXC, ZK, XM: Manuscript editing. All authors read and approved the final manuscript.

**Data curation:** Jingming Wang.

**Formal analysis:** Jingming Wang.

**Methodology:** Xiuchun Yu, Kai Zheng.

**Project administration:** Xiuchun Yu.

**Resources:** Xiuchun Yu.

**Supervision:** Kai Zheng, Ming Xu.

**Writing – original draft:** Jingming Wang.

**Writing – review & editing:** Ming Xu.
